# The Stability of Phenolic Compounds in Fruit, Berry, and Vegetable Purees Based on Accelerated Shelf-Life Testing Methodology

**DOI:** 10.3390/foods12091777

**Published:** 2023-04-25

**Authors:** Kärt Saarniit, Hanna Lang, Rain Kuldjärv, Oskar Laaksonen, Sirli Rosenvald

**Affiliations:** 1 Center of Food and Fermentation Technologies, Mäealuse 2/4, 12618 Tallinn, Estonia; 2 Institute of Chemistry and Biotechnology, Tallinn University of Technology, Akadeemia tee 15, 12618 Tallinn, Estonia; 3 Food Sciences, Department of Life Technologies, Faculty of Technology, University of Turku, 20014 Turku, Finland

**Keywords:** total phenolic content, Folin–Ciocalteu assay, pasteurized purees, packaging, accelerated shelf-life test

## Abstract

Evaluating the stability of polyphenols in fruit, berry, and vegetable purees helps to assess the quality of these products during storage. This study aimed to (1) monitor the stability of total phenolic content (TPC) in four-grain puree with banana and blueberry (FGBB), mango-carrot-sea buckthorn puree (MCB), and fruit and yogurt puree with biscuit (FYB); (2) study the effect of aluminum-layered vs. aluminum-free packaging on the changes in TPC; and (3) assess the suitability of accelerated shelf-life testing (ASLT) methodology to evaluate the stability of polyphenols. The samples were stored at 23 °C for 182, 274, 365, and 427 days. The corresponding time points during ASLT at 40 °C were 28, 42, 56, and 66 days, calculated using Q_10_ = 3. The TPC was determined with Folin–Ciocalteu method. The results revealed that the biggest decrease in TPC took place with high-pH FGBB, which contained fewer ingredients with bioactive compounds. Minor changes were seen in FYB and MCB, which had lower pH values, and contained a larger amount of ingredients that include polyphenols. In addition, the choice of packaging material did not affect the TPC decrease in each puree. Finally, it was concluded that the ASLT methodology is suitable for studying the TPC changes in such purees, but the corresponding Q_10_ factors may vary and should be determined based on the chemical profile and ingredient list of the product.

## 1. Introduction

Consumption of fruits, berries, and vegetables in regular and sufficient amounts is one of the widely approved key parts of achieving a well-balanced diet. In addition to providing vital dietary fiber, fruits, berries, and vegetables also consist of health-beneficial bioactive compounds such as antioxidants and polyphenols [1]. However, the amount of these compounds in food can decrease with applying heat during processing [2], for example, when producing pasteurized purees [3]. In addition, it has been stated that storage time also has a significant effect on the quantity of polyphenols [4,5]. Therefore, it is important to evaluate the stability of phenolic compounds in pasteurized purees to assess the quality of these long-shelf-life products during storage.

Fruit, berry, and vegetable purees are considered good sources of bioactive compounds [6,7]. These compounds include polyphenols, which are secondary metabolites synthesized by plants. Polyphenols have physiological and morphological importance for plants, protecting them against UV radiation, mechanical damage, and microbial infection [8]. Various plants contain different amounts of polyphenols, depending on the genetic background, growing, and climatic conditions [9,10]. For example, blueberries (*Vaccinium* ssp.), sea buckthorn (*Hippophae rhamnoides* L.), and raspberries (*Rubus idaeus* L.) are widely known to contain high amounts of health-beneficial phenolic compounds [7,11,12]. The total content of polyphenols in blueberries ranges from 48–302 mg/100 g of fresh fruit weight [7] and they are considered important to monitor during the shelf-life of blueberry products [13]. More specifically, blueberries contain bioactive compounds such as anthocyanins, flavonols, and phenolic acids [14,15,16]. In addition, raspberries are a good source of anthocyanins [17]. Sea buckthorn, also named as seaberry, has excellent antioxidant properties, mainly due to its high total phenolic content (TPC) which can range from 11–964 mg GAE/100 g [9,12]. Furthermore, fruits contain phenolic compounds that form an important part of their chemical quality. For example, bananas consist of phenolic acids and flavonoids. The TPC of bananas varies in the range of 7–475 mg GAE/100 g, depending on different cultivars and growing conditions [10]. Mango, a popular exotic fruit, also contains various phenolics such as phenolic acids, flavonols, and anthocyanins [18,19]. As well as phenolic-rich berries and fruits, vegetables and cereals are also consumed as health-beneficial sources of bioactive compounds. For instance, the main phenolic compounds in carrots are phenolic acids [20]. In cereals, the TPC depends on the grain type, growing, and processing conditions [21]. For example, the TPC in wheat bran fractions is 750–1082 mg GAE/100 g [22].

Several methods are used to assess the TPC in different matrixes. For example, Folin–Ciocalteu (F–C) assay is one of the most widely used methods to quantify total polyphenols in fruit juices and beverages. In this method, a redox reaction between the F–C phenol’s reagent and the reducing compounds in the sample takes place. However, this assay has its limitations as the reagent not only reacts with polyphenols but also with other compounds in the sample, including ascorbic acid, reducing sugars, SO_2_, etc. As a result, these compounds are also unintentionally quantified as polyphenols. In addition, it is suggested that the F–C assay gives an overview of the total antioxidant capacity and, as phenolics represent antioxidants in most plants, it shows an approximate TPC as a result. Nevertheless, the F–C assay can still be used to evaluate the TPC of samples when polyvinylpolypyrrolidone (PVPP) treatment is applied to separate polyphenolic and non-polyphenolic compounds [23]. Therefore, the F–C assay is still widely implemented to measure the TPC in most food samples. In addition, it also has some considerable benefits compared to other similar methods. For example, the F–C assay does not require an overnight incubation time for the preparation of reagents and the results are easily repeatable. This makes the straightforward method simple to perform, and, in addition, it is inexpensive [24,25].

As the market share of fruit, berry, and vegetable purees is continuously increasing [26], these healthy and convenient products with a high content of bioactive compounds must reach consumers in the best possible condition. In addition, as consumers are becoming more aware of sustainable packaging, industries must provide it to keep up with the demand, ensuring there is no loss in product quality and storage time [27]. However, as the purees need barriers against light and gases to maintain their quality during storage [28,29], packages including aluminum are still often used. Aluminum provides excellent barriers and, at the same time, is stable over a wide range of temperatures, not generating toxic releases when exposed to food. On the other hand, the production of aluminum is an environmentally burdensome process. For example, the global warming potential (GWP) of a metalized PET is 0.197 kg CO_2_ equivalent [30], while the GWP of a pure PET bottle is 32% less burdensome [31]. In addition, the GWP of the aluminum foil itself is 0.382 kg CO_2_ equivalent [30]. Therefore, removing the aluminum layer from multilayered packaging has a positive effect on the environment and it also makes the recycling of the package easier [32]. Regarding barrier needs, aluminum can be replaced with plastics which provide similar barriers against oxygen, water vapor, or gases. For example, oriented polyamide (OPA) is often used as a good oxygen barrier material (OTR = 18 cm^3^/m^2^/day) [33].

To monitor important quality attributes during shelf-life and to determine the end of the storage time of food products efficiently, there is demand from the food industry for fast and reliable methods [34,35]. For this purpose, an accelerated shelf-life test (ASLT) could be used. ASLT allows for the acceleration of the quality deterioration of food products without changing the order of reactions taking place in standard conditions [36]. This methodology enables the faster launch of long shelf-life food products to the market [37]. Furthermore, this approach can be used to evaluate the effect of changes made in the recipe, technology, and/or packaging materials on the quality of the product during storage [35]. The chemical and physical reactions occurring in the product during shelf-life are accelerated by changing storage conditions [38]. These include temperature, oxygen, light, and relative humidity. A higher storage temperature is the most frequently used factor as it affects the kinetics of the reactions the most [37]. The methodology of ASLT is based on the Arrhenius equation (Equation (1)), which shows the effect of temperature on the reaction rate:

(1) $$k=k_{0}\times e^{-\frac{E_{a}}{RT}}$$

where *k* is the kinetic rate constant, *k*_0_ is the exponential factor representing the collision frequency of the reacting molecules, *E_a_* is the activation energy (J), *R* is the universal gas constant (8.3144 J/mol·K), and *T* is the absolute temperature (K) [37,39].

Based on the reaction rates of chemical or physical processes in the product, it is possible to find the acceleration factor Q_10_, which is defined as the number of times a reaction rate changes with a 10 °C change in temperature [40]. Each quality process has its characteristic Q_10_ factor [40] which is calculated to conduct reliable ASLTs [41]. The Q_10_ factor is derived from the Arrhenius equation as follows (Equation (2)):

(2) $$Q_{10}={\left(\frac{k_{2}}{k_{1}}\right)}^{\frac{10}{T_{2}-T_{1}}}$$

where *k*_1_ and *k*_2_ represent the reaction rate at temperatures *T*_1_ and *T*_2_ [42].

The storage stability of phenolic compounds in berry, fruit, and vegetable products has been previously studied by several authors. For example, Srivastava et al. [43] studied the effect of different conditions on blueberry extract. The results showed that the effect of storage was smaller than the impact of the thermal treatment. In addition, Castrejón et al. [44] showed a decrease in the content of total phenols, antioxidants, and anthocyanins in blueberries even during ripening. Patras et al. [45] investigated the decrease and kinetic modeling of anthocyanins in strawberry jam during storage. In this case, the degradation of anthocyanins followed first-order kinetics where the reaction rate increased with an increase in the temperature. The same result was shown by Celli et al. [46] who studied the degradation of anthocyanins in freeze-dried microencapsulates from lowbush blueberries. Andersson et al. [47] studied the effect of storage time and temperature on the stability of TPC in beverages prepared with sea buckthorn berry puree, concluding that the largest decrease in TPC occurred immediately after production and later no significant change was observed. However, as far as the authors know, there is no clear information in the literature about the suitability of ASLT methodology for fruit, berry, and vegetable purees.

Therefore, the aims of this study were to (1) monitor the stability of phenolic compounds in different fruit, berry, and vegetable purees; (2) study the effect of AL-layered vs. AL-free packaging on the changes in TPC; and (3) assess the suitability of accelerated shelf-life testing methodology to assess the changes in phenolic content in these purees.

## 2. Materials and Methods

### 2.1. Purees and Packaging

The samples of packaged purees were supplied by a local producer (Salvest AS, Tartu, Estonia). Three different organic purees were used in this study: four-grain puree with banana and blueberry (FGBB), mango-carrot-sea buckthorn puree (MCB), and fruit and yogurt puree with biscuit (FYB). The ingredients and nutritional compositions of 100 g of purees, labeled on the packages, are given in Table 1.

The purees were packaged in two types of doypack pouches where one version included an aluminum (AL) layer and the other one was AL-free (Gualapack S.p.A, Castellazzo Bormida, Italy). In addition, the doypacks included materials like polyethylene terephthalate (PET), oriented polyamide (OPA), and polypropylene (PP). In more detail, the doypack composition with AL-layer consisted of 12 µm PET/9 µm ALU/15 µm OPA/75 µm PP. The doypack without the AL-layer consisted of 12 µm PET/15 µm OPA/70 µm PP. The spout material of both packages was PP. The oxygen and water vapor transmission rates for both doypacks were <1 cm^3^/m^2^/24 h and <1 g/m^2^/24 h, respectively.

The packaged purees were heat treated with the internal temperature being 108 °C for 31 min for FGBB, 103 °C for 43 min for MCB, and 103 °C for 17 min for FYB.

### 2.2. Reagents and Standards

Folin–Ciocalteu reagent (Sigma-Aldrich, Taufkirchen, Germany), gallic acid monohydrate (Sigma-Aldrich, Germany), sodium bicarbonate (Sigma-Aldrich, Germany), poly(vinylpolypyrrolidone) (PVPP) (Sigma-Aldrich, Germany), acetone (Sigma-Aldrich, Germany).

### 2.3. Design of Shelf-Life Tests

Purees packaged in doypacks were stored in a carton board box at room temperature (23 °C) and in a climate chamber at 40 °C and 50% of relative humidity (Memmert UN750, Büchenbach, Germany). The samples were stored in the dark to simulate the most likely condition applied in the warehouse. As the expected shelf-lives of the samples at room temperature were 12 months, the testing time points were chosen based on this to describe possible changes taking place before and after the expected end of storage. Therefore, the testing points for room temperature storage were 0-point (immediately after production), 182 days (6 months), 274 days (9 months), 365 days (12 months), and 427 days (14 months). For the ASLT at 40 °C, the storage time for each corresponding analysis point was calculated, taking into account the Q_10_ factor (Equation (3)).

(3) $$Accelerated\;aging\;time\;\left(AAT\right)=\frac{Desired\;real\;time\;\left(RT\right)}{Q_{10}{}^{\left(\frac{T_{AA}-T_{RT}}{10}\right)}}$$

where *AAT* is the accelerated aging time at accelerated aging temperature (*T_AA_*) and *RT* is the real storage time at real storage time temperature (*T_RT_*) [48].

As the literature states, the Q_10_ for almost all food products is approximately 3 [49]. Therefore, the time points of the ASLT which corresponded to 6, 9, 12, and 14 months in room temperature storage were calculated to be 28, 42, 56, and 66 days at 40 °C. At each time point, 2 sample replicates from both storage conditions were taken for analysis.

### 2.4. pH Analysis

The pH was measured using a pH-meter (Mettler-Toledo International Inc., Columbus, OH, USA). The analysis was done by inserting a pH-electrode into a previously homogenized sample. Two measurements were performed for both sample replicates.

### 2.5. Analysis of Total Phenolic Content

The content of total phenolic compounds was determined using the F–C method as described by Yap et al. [50]. The extraction of total phenolic compounds from different food matrices was performed as described by Sulaiman et al. [51] with some modifications, and the polyvinylpolypyrrolidone (PVPP) treatment was performed as described by Bridi et al. [23] with some modifications.

Briefly, 1 g of sample was weighed into 15 mL high-speed centrifuge tubes, 5 mL of 70% acetone was added, and mixed thoroughly. Sample extracts were rotated for 60 min using rotator Stuart SB3 (TEquipment, Long Branch, NJ, USA). Sample extracts were centrifuged (21,000× *g* at 18 °C for 10 min), filtered with a microfilter (CHROMAFIL Xtra PET-20/13, 0.2 µm), and diluted with acetone up to 5 times, if necessary.

To separate polyphenols and non-polyphenolic derivatives (sugars, ascorbic acid, and sulfite) from the samples and, therefore, to see the amount of interfering compounds, a pretreatment with polyvinylpolypyrrolidone (PVPP) was used. The PVPP was suspended in milliQ water and was well shaken before being added to the filtered sample. PVPP treatment was applied as follows: 0.5 mL of PVPP suspension (40 g/L) was added to 0.5 mL of filtered sample and the mixture was rotated for 10 min using rotator Stuart SB3 (TEquipment, USA). After PVPP treatment, the samples were centrifuged (21,000× *g* at 18 °C for 10 min), filtered with a microfilter (CHROMAFIL Xtra PET-20/13, 0.2 µm), and diluted with acetone up to 5 times, if necessary.

The content of TPC was determined using the F–C method. For this, 20 µL of filtered and diluted sample extracts (PVPP treated or untreated) were mixed with 100 µL of 0.2N F–C reagent. After 5 min, the reaction was stopped by adding 80 µL of 7.5% sodium carbonate solution. After stopping the reaction, the samples were incubated for 30 min at 37 °C. After incubation, the absorbance of the samples was measured with a BioTek Synergy H1 multi-mode microplate reader (Agilent Technologies, Inc., Santa Clara, CA, USA) at a wavelength of 765 nm. A 70% acetone solution was used as an absorbance blank. The results were expressed as gallic acid equivalent (GAE). TPC of the sample was calculated as a difference between before and after PVPP pretreatment in mg/g of the gallic acid equivalent (mg GAE/g).

### 2.6. Statistical Analysis

Statistical significance testing was performed in R 4.2.2 (The R Foundation for Statistical Computing, Vienna, Austria) using package “emmeans” 1.8.3. The TPC response variable was modeled by a cubic B-spline of time data plus packaging. The model fit was evaluated visually and using the adjusted coefficient of determination. The significances were calculated across time points and packaging for each different product and each different storage temperature by using pairwise t-test comparisons between the estimated marginal means. The confidence level was set to 0.95 and adjusted using the Bonferroni method. *p*-values were adjusted according to the Benjamini–Hochberg method [52]. The significance level for compact letter displays was set to 0.05.

## 3. Results and Discussion

### 3.1. pH

The initial pH values of the purees differed marginally. For example, in the 0-point, MCB had the lowest pH value with 3.9 in both packages. The initial pH of FYB was similar, with the value being 4.0 also in both doypacks. However, FGBB with fewer acidic ingredients showed the highest pH value of 4.8 in both packages. During both storage tests, the pH values of the purees did not change.

### 3.2. Effect of Packaging Material on the Changes in TPC

The results of initial TPCs from each puree at 0-point showed that there were no statistical differences, whether the puree was packaged in AL-layered or AL-free packaging. However, there were slight distinctions during the storage tests. For example, when the decrease of TPC in FGBB puree was slightly bigger in AL-free packaging at both 23 °C and 40 °C, the results were the opposite for the FYB puree in which the TPC decreased faster in AL-layered packaging at both storage temperatures.

**Figure 1:**
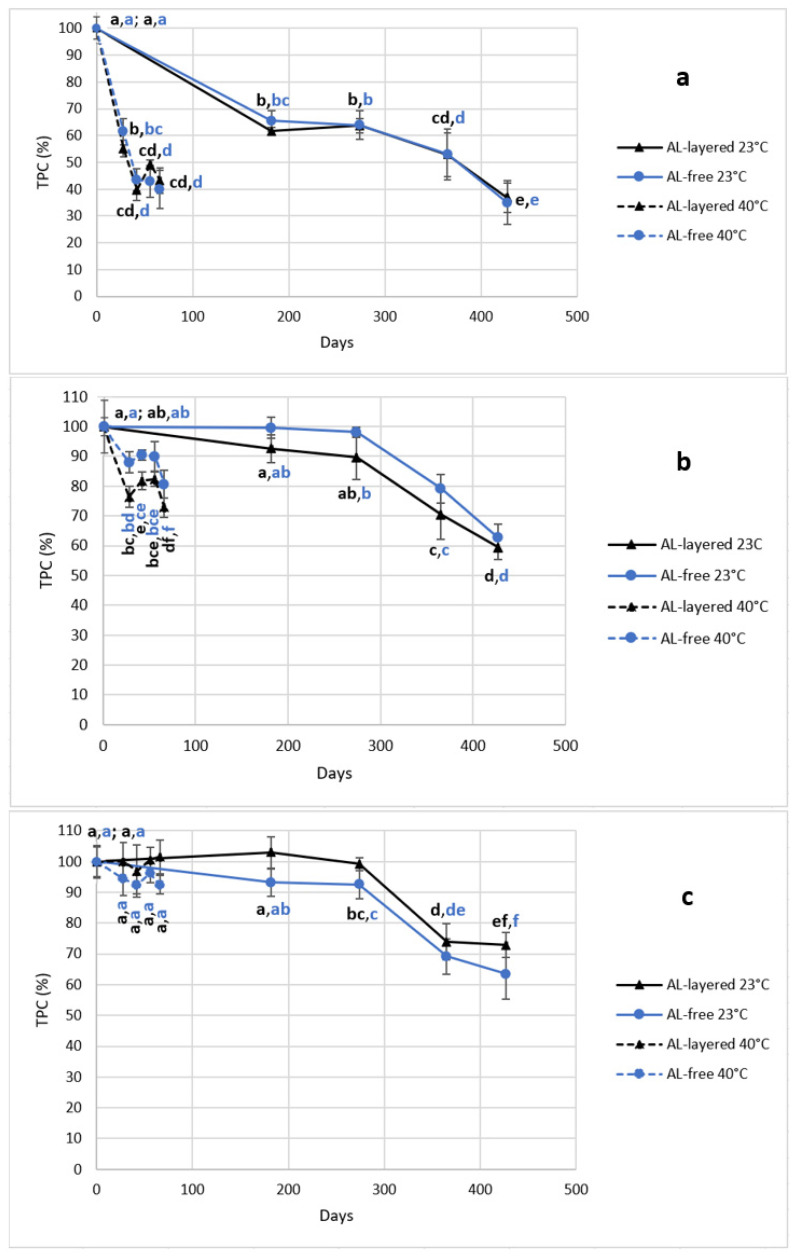
TPC changes in FGBB (**a**), FYB (**b**), and MCB (**c**), at 23 °C and 40 °C. The significances are calculated across time points and packaging for each different product and each different storage temperature by using pairwise *t*-test comparisons between the estimated marginal means. The 0-point results are statistically different for FYB (**b**), where 23 °C = group ab, ab, 40 °C = group a, a.

In more detail, by the end of the tests, the phenolic content in FGBB puree decreased by 65% and 60% in AL-free packaging at 23 °C and 40 °C, respectively, while in AL-layered doypacks, the decrease was 63% at 23 °C and 57% at 40 °C (Figure 1a). On the other hand, the TPC of FYB puree decreased by 41% in AL-layered packaging at the 23 °C storage test and 27% during the ASLT. In AL-free packaging, the decrease in FYB was 37% at room temperature and 19% at 40 °C storage (Figure 1b). In addition, the decrease of TPC in the MCB puree, packaged in AL-free packaging and stored at 23 °C, was also slightly bigger (37%), while in AL-layered packaging, the decrease was 27% in the same storage conditions (Figure 1c). However, as seen from Figure 1 and Supplementary Materials (Table S1), there were no statistical differences between the results at each time point during both storage tests, whether the purees were packaged in AL-layered or AL-free doypacks.

It is known that aluminum is used to enhance packaging materials with its good barriers, including providing a high barrier against light [33]. At the same time, it has been shown that the storage stability of polyphenols can be easily affected by light from the surrounding environment [53]. Therefore, it is concluded that fruit, berry, and vegetable purees should be packaged in opaque materials. However, the storage tests in the current experiment were conducted in environments without light and, as no statistical differences were seen based on the results, it is concluded that in this case, the packaging choice did not affect the decrease in the TPC.

### 3.3. Comparison of Changes in TPC of Three Purees

The highest content of phenolics in the samples before storage was found in the FYB and MCB purees (Supplementary Materials, Table S1). The FYB contained mainly banana, mango, and raspberry, which are known to have high contents of phenolic compounds [10,54,55]. For example, the initial content of TPC in FYB was 49.8 mg GAE/100 g in AL-layered doypack, and 50.0 mg GAE/100 g in AL-free packaging (Supplementary Materials, Table S1). The MCB puree also showed the high phenolic content of mango, carrot, and sea buckthorn [55,56,57]. For this puree, the initial contents of TPC were 51.7 mg GAE/100 g and 53.0 mg GAE/100 g for AL-layered and AL-free doypacks, respectively. At the same time, FGBB showed the lowest initial content of TPC with 30.9 mg GAE/100 g and 29.2 mg GAE/100 g in doypacks with AL and without AL-layer, respectively. This is probably because while the FYB and MCB purees contained almost 100% fruits, berries, and/or vegetables, FGBB consisted of only 45% of ingredients with phenolic content.

In more detail, the biggest changes in the TPC during both storage tests took place with FGBB, in which a rapid change already occurred in the first time point (Figure 1a). This puree contained the lowest amount of fruits, berries, or vegetables and also had the highest pH compared to other purees. Previously, it has been argued by Narita et al. [58] that phenolic compounds are more stable under acidic pH conditions, which in part explains why the decrease of TPC in FGBB was significantly bigger during both storage tests, compared to the FYB and MCB purees. However, Friedman [2] has also stated that the stability of phenolic compounds not only depends on pH but also on the structure of the specific compound. For example, the FGBB puree consisted of 30% banana puree, 8% blueberry puree, and 7% four-grain cereals. According to the literature, these ingredients contain various polyphenols which may have different stabilities during storage. For example, bananas and four-grain cereals can contain ferulic acid [10], which may degrade during storage due to its carboxyl group [59]. However, ferulic acid may also be bound to other compounds in the matrix, such as reducing sugars or other polymeric structures [60], making the phenolic component more stable during storage [61]. Next to banana, FGBB also consisted of blueberries, which, according to the literature, may contain chlorogenic acid, which can be stable in acidic conditions and high temperatures [16]. In addition, blueberries may include stable glycolysed flavonols such as quercetin-based and myricetin-based galactosides and glucosides [14,62]. However, blueberries can also contain anthocyanins such as cyanidins, which are sensitive to higher temperatures [15] but are more stable at lower pHs [63]. Therefore, the possible anthocyanins in FGBB may be less stable due to the high pH of the matrix. In addition, anthocyanins can also exist in unstable free forms when the matrix has undergone processes such as heat treatment or storage at ambient temperatures. On the other hand, it has been stated by Mäkilä et al. [64] that otherwise relatively unstable anthocyanins can form more stable compounds during storage through polymerization, co-pigmentation with other phenolic compounds, and further degradation to hydroxybenzoic acids. Based on this discussion, it is hypothesized that the FGBB puree contained ingredients that may have included polyphenols with different stability throughout storage. However, the FGBB puree had the highest pH, consisting of nearly 50% of water, making the matrix the least favorable for phenolics to maintain stability during storage.

Similarly to FGBB, the FYB puree also contained banana as the main ingredient. In more detail, FYB consisted of 37% banana puree, 36% mango puree, 10% raspberry puree, and 2% four-grain cereals. Overall, this product had a lower pH throughout storage and showed a smaller decrease in TPC than FGBB in both storage conditions (Figure 1b). Similarly to the FGBB puree, it is hypothesized that FYB could also contain ferulic acid originating from bananas and four-grain cereals [59]. However, in addition to this tropical fruit, FYB also contained mango in a large proportion. For example, based on the literature, mango may include various phenolics such as phenolic acids, flavonols, and anthocyanins [18,19]. While gallic acid and quercetin derivatives in mango may be more stable [15,16,63], anthocyanins and catechins might degrade more easily during storage [15,18]. As well as these components, FYB also contained raspberries in smaller quantities. These acidic berries can contain cyanidins and ellagitannins, which are both temperature-sensitive compounds [15,17]. Based on this discussion, it is concluded that the FYB puree may have contained both stable and less stable bioactive compounds. However, the puree included more fruits and berries than FGBB, and therefore the pH of the matrix was also lower, which might have contributed to the compounds being more stable during storage.

Finally, the smallest changes in TPC during storage were seen in the MCB puree (Figure 1c) with the lowest pH. Similarly to FYB, this product also contained mango but in a larger amount. More specifically, the MCB puree consisted of mango (52%), carrot (40%), and sea buckthorn (8%). Based on the literature, it is speculated that this puree may contain possibly stable phenolic acids and flavonoids that might be originating from the mango [15,16,18,20]. However, the puree may also contain compounds in smaller quantities that may be less stable, for example, catechins [15,18,65] from mango and sea buckthorn and salicylic acid from sea buckthorn [66]. Based on this discussion, it is hypothesized that MCB may also have contained both stable and less stable phenolic compounds. However, even more importantly, the MCB puree consisted only of fruits, berries, and vegetables and had the lowest pH, which may have created the most advantageous environment for keeping the bioactive compounds stable throughout storage.

### 3.4. Effect of Storage Temperature and Following Linear Regression Models of TPC Changes

Overall, the biggest decreases in the TPC in each puree occurred during storage at 23 °C (Figure 1, Supplementary Materials, Table S1). However, there are considerable differences when comparing the results between purees at both storage temperatures. For example, with the FGBB puree, the decrease during storage at 23 °C was 63% in the AL-layered doypack and 65% in AL-free packaging. In comparison, by the end of the ASLT, the TPC decreases in the FGBB puree were slightly smaller, with 57% in AL-layered and 60% in AL-free packaging (Figure 1a).

However, for the other purees, the results were not as similar in a comparison of different storage temperatures. For instance, while the TPC in the FYB puree decreased by 41% and 37% by the end of the room temperature storage test in AL-layered and AL-free packaging, during storage at 40 °C, the decrease in both packaging materials was only 27% and 19%, respectively (Figure 1b). Furthermore, during the ASLT, no significant changes in the TPC were found in the MCB puree in either packaging. However, during the storage at 23 °C, the TPC decrease in the same puree was more similar to FYB, with 27% in AL-layered and 37% in AL-free packaging (Figure 1c).

Therefore, the storage temperature had different effects on the total phenolic content based on the puree and its properties. As stated in the literature [2,58], phenolic compounds are more stable under acidic pH conditions, and the stability of phenolics depends on the specific compound and its structure. The experiment results are in accordance with the literature, showing that the phenolic content is more stable in matrixes with a lower pH. In addition, the literature [45,46] states that the rate of degradation of phenolic compounds increases with an increase in the temperature. However, there were minor or no changes in the FYB and MCB purees during the ASLT, showing that the ingredients in these purees may have contained compounds which could be more stable at higher storage temperatures.

**Figure 2:**
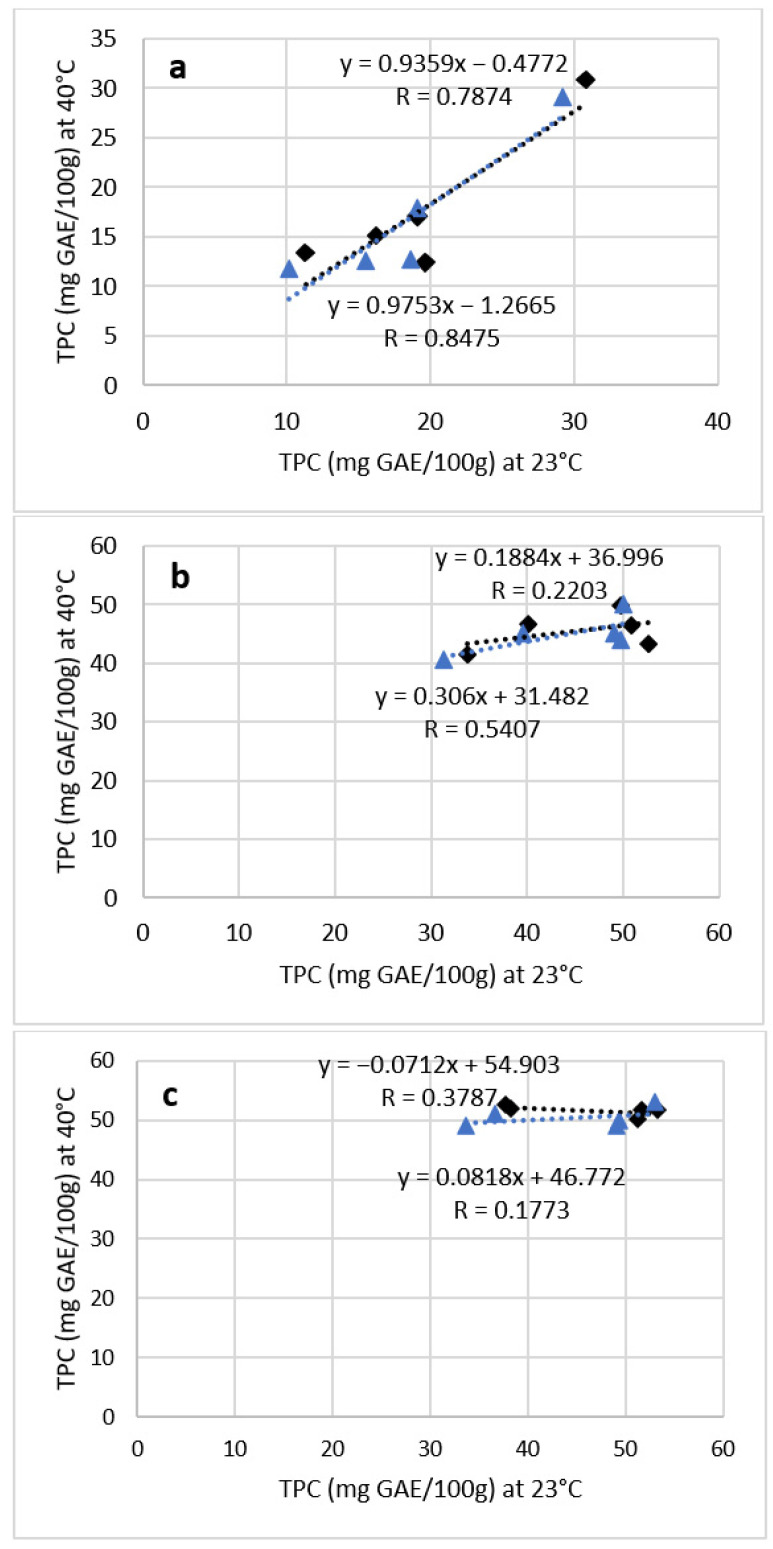
Linear regression models of TPC in FGBB (**a**), FYB (**b**), and MCB (**c**) at 23 °C and 40 °C (rectangle = AL-layered, triangle = AL-free).

Considering the previous discussion, linear regression models of the changes in the TPC can be presented based on the effect of storage temperatures. This is necessary in order, in the future, to conduct accurate ASLTs with similar purees. To assess whether the chosen acceleration factor (Q_10_ = 3) is suitable for conducting an ASLT with each puree, the correlation coefficients of TPC at both storage temperatures are represented on Figure 2.

Unlike for the MCB and FYB purees, the comparative changes in the TPC between 23 °C and 40 °C storage tests were most similar for the FGBB puree (Figure 2a). For example, with FGBB in AL-layered packaging, the correlation coefficient for the decrease in TPC between both storage temperatures was 0.7874. In AL-free packaging, the coefficient was even higher. Since FGBB had the highest pH and consisted of nearly 50% water, it also might have had the least favorable matrix for phenolics to maintain stability during storage. Therefore, it was clearly seen that the changes in both storage conditions took place rapidly and comparably. Although the TPC decrease at room temperature in the FGBB puree was still slightly larger (by 5–6%) than during ASLT, it can be said that the chosen acceleration factor (Q_10_ = 3) is suitable for conducting an ASLT with the FGBB puree.

For the FYB puree, the decrease in TPC during the ASLT was approximately 1.5 and 2 times smaller for AL-layered and AL-free packaging, respectively, compared to room temperature storage. This difference is also reflected in the regression model between the results of both storage conditions. The correlation coefficients of the TPC in the FYB purees packaged in AL-layered and AL-free doypacks at 23 °C and 40 °C are remarkably lower than for the FGBB puree (Figure 2b). As the changes in FYB at a higher storage temperature were smaller, it can be said that the chosen acceleration factor (Q_10_ = 3) was overrated for the FYB puree and was initially expected to reflect a faster decrease than actually took place. Therefore, it is concluded that to conduct an accurate ASLT with FYB, the acceleration factor should be smaller than Q_10_ = 3.

No significant changes in TPC took place with MCB at 40 °C throughout the ASLT, showing that the ingredients in this puree may have contained compounds which could be more stable at a higher storage temperature. In addition, the decrease was also the smallest at room temperature storage, compared to other purees. This outcome is also reflected in the regression models where the coefficient for MCB in AL-layered packaging was 0.3787, and for AL-free packaging it was even lower (Figure 2c). As no significant changes took place during the ASLT, it is concluded that the chosen Q_10_ factor (Q_10_ = 3) was also overrated for the MCB puree. Therefore, this product requires the use of a smaller acceleration factor and, consequently, a longer period of storage time at 40 °C to reflect the changes taking place during real-time storage.

## 4. Conclusions

The results revealed that the stability of phenolic compounds in different fruit, berry, and vegetable purees is product specific, mostly depending on the ingredients used, the amount of these ingredients, and the pH. The most significant decrease in TPC took place with the FGBB puree, with the highest pH and the smallest amount of ingredients containing phenolic compounds. On the other hand, minor changes in TPC took place with the FYB and MCB purees. It was discussed that the FYB puree may have contained both stable and less stable bioactive compounds. However, the pH of the matrix was also lower, which might have contributed to the compounds being more stable during storage. The smallest decrease in TPC was found for the MCB puree. In this case, the puree consisted only of fruits, berries, and vegetables and had the lowest pH, which may have created the most advantageous environment for keeping the bioactive compounds stable throughout storage.

The results showed that the choice of packaging material did not affect the TPC decrease in each puree. However, this conclusion assumes that the products are mostly stored in dark conditions throughout the supply chain. Therefore, the effect of AL-layered vs. AL-free packaging on the content of total phenols may be different when conducting the tests in environments exposed to light.

To study the suitability of ASLT methodology for assessing the phenolic content changes in these purees, linear regression models were presented. The results showed that the decrease in TPC during 23 °C and 40 °C storage tests was most similar for the FGBB puree. This was also reflected in the highest correlation coefficients for the FGBB puree samples. On the other hand, the correlation coefficients for FYB were lower due to smaller changes during ASLT. With the MCB puree, no significant changes in the TPC took place at 40 °C, resulting in the lowest correlation coefficients. Therefore, the chosen acceleration factor (Q_10_ = 3) was suitable for the FGBB puree but not for the FYB and MCB purees. As a result, it is concluded that the ASLT methodology is suitable for studying the TPC changes in such purees, but the corresponding Q_10_ factors may vary and should be determined based on the chemical profile and ingredient list of the product.

## Figures and Tables

**Table 1:** The nutritional composition of four-grain puree with banana and blueberry (FGBB), mango-carrot-sea buckthorn (MCB), and fruit and yogurt puree with biscuit (FYB) in 100 g of product.

Nutritional Composition	FGBB	MCB	FYB
Ingredients	Water, banana puree (30%), blueberry puree (8%), four-grain cereals (rye, oat, wheat, barley) (7%), and rapeseed oil	Mango puree (52%), carrot puree (40%), and sea buckthorn puree (8%)	Banana puree (37%), mango puree (36%), yogurt (15%), raspberry puree (10%), and biscuit (2%) including wheat flour, butter, and water
Energy (kcal/kJ)	62/262	62/262	88/368
Fat (g/100 g)	1.4	0.0	1.3
Unsaturated Fatty Acids (g/100 g)	0.0	0.0	0.8
Carbohydrates (g/100 g)	11.0	13.0	16.0
Sugars (g/100 g)	3.8	11.0	11.0
Protein (g/100 g)	1.2	0.6	1.3
Salt (g/100 g)	0.0	0.0	0.0

## Data Availability

The data presented in this study are available on request from the corresponding author.

## Supplementary Materials

The following supporting information can be downloaded at: https://www.mdpi.com/article/10.3390/foods12091777/s1, Table S1. Changes in TPC (mg GAE/100 g) of four-grain puree with banana and blueberry (FGBB), mango-carrot-sea buckthorn puree (MCB), and fruit and yogurt puree with biscuit (FYB) during storage tests at 23 °C and 40 °C.

## References

[1] Slavin J.L., Lloyd B. (2012). Health Benefits of Fruits and Vegetables. Adv. Nutr. Int. Rev. J..

[2] Friedman M. Effects of Food Processing. http://www.wheatfoods.org.

[3] Cano-Lamadrid M., Artés-Hernández F. (2022). Thermal and Non-Thermal Treatments to Preserve and Encourage Bioactive Compounds in Fruit- and Vegetable-Based Products. Foods.

[4] Salazar-Orbea G.L., García-Villalba R., Bernal M.J., Hernández A., Tomás-Barberán F.A., Sánchez-Siles L.M. (2023). Stability of phenolic compounds in apple and strawberry: Effect of different processing techniques in industrial set up. Food Chem..

[5] Zhang Y., Truzzi F., D’amen E., Dinelli G. (2021). Effect of Storage Conditions and Time on the Polyphenol Content of Wheat Flours. Processes.

[6] Govers C., Kasikci M.B., Van Der Sluis A.A., Mes J.J. (2018). Review of the health effects of berries and their phytochemicals on the digestive and immune systems. Nutr. Rev..

[7] Michalska A., Łysiak G. (2015). Bioactive Compounds of Blueberries: Post-Harvest Factors Influencing the Nutritional Value of Products. Int. J. Mol. Sci..

[8] Siracusa L., Ruberto G. (2014). Plant polyphenol profiles as a tool for traceability and valuable support to biodiversity. Polyphenols in Plants: Isolation, Purification and Extract Preparation.

[9] Korekar G., Dolkar P., Singh H., Srivastava R.B., Stobdan T. (2014). Variability and the genotypic effect on antioxidant activity, total phenolics, carotenoids and ascorbic acid content in seventeen natural population of Seabuckthorn (*Hippophae rhamnoides* L.) from trans-Himalaya. LWT.

[10] Singh B., Singh J.P., Kaur A., Singh N. (2016). Bioactive compounds in banana and their associated health benefits—A review. Food Chem..

[11] Frías-Moreno M.N., Parra-Quezada R.A., González-Aguilar G., Ruíz-Canizales J., Molina-Corral F.J., Sepulveda D.R., Salas-Salazar N., Olivas G.I. (2021). Quality, Bioactive Compounds, Antioxidant Capacity, and Enzymes of Raspberries at Different Maturity Stages, Effects of Organic vs. Conventional Fertilization. Foods.

[12] Ren R., Li N., Su C., Wang Y., Zhao X., Yang L., Li Y., Zhang B., Chen J., Ma X. (2020). The bioactive components as well as the nutritional and health effects of sea buckthorn. RSC Adv..

[13] Martynenko A., Chen Y. (2016). Degradation kinetics of total anthocyanins and formation of polymeric color in blueberry hydrothermodynamic (HTD) processing. J. Food Eng..

[14] Chaaban H., Ioannou I., Chebil L., Slimane M., Gérardin C., Paris C., Charbonnel C., Chekir L., Ghoul M. (2017). Effect of heat processing on thermal stability and antioxidant activity of six flavonoids. J. Food Process. Preserv..

[15] Esparza I., Cimminelli M.J., Moler J.A., Jiménez-Moreno N., Ancín-Azpilicueta C. (2020). Stability of Phenolic Compounds in Grape Stem Extracts. Antioxidants.

[16] Friedman M., Jürgens H.S. (2000). Effect of pH on the Stability of Plant Phenolic Compounds. J. Agric. Food Chem..

[17] Sójka M., Janowski M., Grzelak-Błaszczyk K. (2019). Stability and transformations of raspberry (*Rubus idaeus* L.) ellagitannins in aqueous solutions. Eur. Food Res. Technol..

[18] Kim H., Castellon-Chicas M.J., Arbizu S., Talcott S.T., Drury N.L., Smith S., Mertens-Talcott S.U. (2021). Mango (*Mangifera indica* L.) Polyphenols: Anti-Inflammatory Intestinal Microbial Health Benefits, and Associated Mechanisms of Actions. Molecules.

[19] Masibo M., He Q. (2008). Major Mango Polyphenols and Their Potential Significance to Human Health. Compr. Rev. Food Sci. Food Saf..

[20] Arscott S.A., Tanumihardjo S.A. (2010). Carrots of Many Colors Provide Basic Nutrition and Bioavailable Phytochemicals Acting as a Functional Food. Compr. Rev. Food Sci. Food Saf..

[21] Ragaee S., Seetharaman K., Abdel-Aal E.-S.M. (2014). The Impact of Milling and Thermal Processing on Phenolic Compounds in Cereal Grains. Crit. Rev. Food Sci. Nutr..

[22] ilić S. (2016). Phenolic Compounds of Wheat. Their Content, Antioxidant Capacity and Bioaccessibility. MOJ Food Process. Technol..

[23] Bridi R., Troncoso M.J., Folch-Cano C., Fuentes J., Speisky H., López-Alarcón C. (2014). A Polyvinylpolypyrrolidone (PVPP)-Assisted Folin–Ciocalteu Assay to Assess Total Phenol Content of Commercial Beverages. Food Anal. Methods.

[24] Everette J.D., Bryant Q.M., Green A.M., Abbey Y.A., Wangila G.W., Walker R.B. (2010). Thorough study of reactivity of various compound classes toward the Folin−Ciocalteu reagent. J. Agric. Food Chem..

[25] Ma S., Kim C., Neilson A.P., Griffin L.E., Peck G.M., O’Keefe S.F., Stewart A.C. (2019). Comparison of Common Analytical Methods for the Quantification of Total Polyphenols and Flavanols in Fruit Juices and Ciders. J. Food Sci..

[26] Grand View Research, “Fruit Puree Market Size, Share & Trends Analysis Report by Product (Tropical & Exotic, Citrus, Berries), by Application (Beverages, Bakery & Snacks, Baby Food), by Region, and Segment Forecasts, 2020–2027,” 2019. https://www.grandviewresearch.com/industry-analysis/fruit-puree-market.

[27] Bauer A.-S., Tacker M., Uysal-Unalan I., Cruz R.M.S., Varzakas T., Krauter V. (2021). Recyclability and Redesign Challenges in Multilayer Flexible Food Packaging—A Review. Foods.

[28] Sonar C.R., Rasco B., Tang J., Sablani S.S. (2019). Natural color pigments: Oxidative stability and degradation kinetics during storage in thermally pasteurized vegetable purees. J. Sci. Food Agric..

[29] Franco R.R., Ojeda G.A., Rompato K.M., Sgroppo S.C. (2021). Effects of short-wave ultraviolet light, ultrasonic and microwave treatments on banana puree during refrigerated storage. Food Sci. Technol. Int..

[30] Bayus J., Ge C., Thorn B. (2016). A preliminary environmental assessment of foil and metallized film centered laminates. Resour. Conserv. Recycl..

[31] Tamburini E., Costa S., Summa D., Battistella L., Fano E.A., Castaldelli G. (2021). Plastic (PET) vs bioplastic (PLA) or refillable aluminium bottles—What is the most sustainable choice for drinking water? A life-cycle (LCA) analysis. Environ. Res..

[32] Schmidt J., Grau L., Auer M., Maletz R., Woidasky J. (2022). Multilayer Packaging in a Circular Economy. Polymers.

[33] Robertson G.L. (2013). Food Packaging: Principles and Practice.

[34] Haouet M.N., Tommasino M., Mercuri M.L., Benedetti F., Di Bella S., Framboas M., Pelli S., Altissimi M.S. (2018). Experimental accelerated shelf life determination of a ready-to-eat processed food. Ital. J. Food Saf..

[35] Mizrahi S. (2004). Accelerated shelf-life tests. Understanding and Measuring the Shelf-Life of Food.

[36] Corradini M.G. (2018). Shelf Life of Food Products: From Open Labeling to Real-Time Measurements. Annu. Rev. Food Sci. Technol..

[37] Calligaris S., Manzocco L., Anese M., Nicoli M.C. (2019). Accelerated shelf life testing. Food Quality and Shelf Life.

[38] Kilcast D., Subramanian P. (2000). Introduction. The Stability and Shelf-Life of Food.

[39] Taoukis P., Giannakourou M. (2004). Temperature and food stability: Analysis and control. Understanding and Measuring the Shelf-Life of Food.

[40] Toledo R.T. (2007). Kinetics of Chemical Reactions in Foods. Fundamentals of Food Process Engineering.

[41] Bravi E., Sileoni V., Perretti G., Marconi O. (2020). Accelerated shelf-life model of gluten-free rusks by using oxidation indices. Food Chem..

[42] Fu B., Labuza T.P. (1997). Shelf-Life Testing: Procedures and Prediction Methods. Quality in Frozen Foods.

[43] Srivastava A., Akoh C.C., Yi W., Fischer J., Krewer G. (2007). Effect of Storage Conditions on the Biological Activity of Phenolic Compounds of Blueberry Extract Packed in Glass Bottles. J. Agric. Food Chem..

[44] Castrejón A.D.R., Eichholz I., Rohn S., Kroh L.W., Huyskens-Keil S. (2008). Phenolic profile and antioxidant activity of highbush blueberry (Vaccinium corymbosum L.) during fruit maturation and ripening. Food Chem..

[45] Patras A., Brunton N., Tiwari B.K., Butler F. (2011). Stability and Degradation Kinetics of Bioactive Compounds and Colour in Strawberry Jam during Storage. Food Bioprocess Technol..

[46] Celli G.B., Dibazar R., Ghanem A., Brooks M.S.-L. (2016). Degradation kinetics of anthocyanins in freeze-dried microencapsulates from lowbush blueberries (*Vaccinium angustifolium* Aiton) and prediction of shelf-life. Dry. Technol..

[47] Andersson S.C., Ekholm A., Johansson E., Olsson M.E., Sjöholm I., Nyberg L., Nilsson A., Rumpunen K. (2015). Effect of storage time and temperature on stability of bioactive compounds in aseptically packed beverages prepared from rose hips and sea buckthorn berries. Agric. Food Sci..

[48] ASTM International Standard Guide for Accelerated Aging of Sterile Barrier Systems for Medical Devices (ASTM F1980-16). https://www.astm.org/f1980-16.html.

[49] Choi J.-Y., Lee H.-J., Cho J.-S., Lee Y.-M., Woo J.-H., Moon K.-D. (2017). Prediction of shelf-life and changes in the quality characteristics of semidried persimmons stored at different temperatures. Food Sci. Biotechnol..

[50] Yap S.K., Chin N.L., Yusof Y.A., Chong K.Y. (2019). Quality characteristics of dehydrated raw *Kelulut* honey. Int. J. Food Prop..

[51] Sulaiman S.F., Sajak A.A.B., Ooi K.L., Supriatno, Seow E.M. (2011). Effect of solvents in extracting polyphenols and antioxidants of selected raw vegetables. J. Food Compos. Anal..

[52] Benjamini Y., Hochberg Y. (1995). Controlling the False Discovery Rate: A Practical and Powerful Approach to Multiple Testing. J. R. Stat. Soc. Ser. B.

[53] Yu L., Wu Y., Liu D., Sheng Z., Liu J., Chen H., Feng W. (2022). The kinetic behavior of antioxidant activity and the stability of aqueous and organic polyphenol extracts from navel orange peel. Food Sci. Technol..

[54] Canadanovic-Brunet J., Vulic J., Cebovic T., Cetkovic G., Canadanovic V., Djilas S., Saponjac V.T. (2017). Phenolic profile, antiradical and antitumour evaluation of raspberries pomace extract from Serbia. Iran. J. Pharm. Res..

[55] Maldonado-Celis M.E., Yahia E.M., Bedoya R., Landázuri P., Loango N., Aguillón J., Restrepo B., Ospina J.C.G. (2019). Chemical Composition of Mango (Mangifera indica L.) Fruit: Nutritional and Phytochemical Compounds. Front. Plant Sci..

[56] Criste A., Urcan A.C., Bunea A., Furtuna F.R.P., Olah N.K., Madden R.H., Corcionivoschi N. (2020). Phytochemical Composition and Biological Activity of Berries and Leaves from Four Romanian Sea Buckthorn (*Hippophae Rhamnoides* L.) Varieties. Molecules.

[57] Leja M., Kamińska I., Kramer M., Maksylewicz-Kaul A., Kammerer D., Carle R., Baranski R. (2013). The Content of Phenolic Compounds and Radical Scavenging Activity Varies with Carrot Origin and Root Color. Plant Foods Hum. Nutr..

[58] Narita Y., Inouye K. (2013). Degradation Kinetics of Chlorogenic Acid at Various pH Values and Effects of Ascorbic Acid and Epigallocatechin Gallate on Its Stability under Alkaline Conditions. J. Agric. Food Chem..

[59] Cheng Y., Xu Q., Liu J., Zhao C., Xue F., Zhao Y. (2014). Decomposition of Five Phenolic Compounds in High Temperature Water. J. Braz. Chem. Soc..

[60] Jankovská P., Čopíková J., Sinitsya A. (2001). The determination of ferulic acid in sugar beet pulp. Czech J. Food Sci..

[61] van Lith R., Ameer G.A., Dziubla T., Butterfield D.A. (2016). Antioxidant Polymers as Biomaterial. Oxidative Stress and Biomaterials.

[62] Lavelli V., Kerr W. (2019). Moisture properties and stability of novel bioactive ingredients. Food Quality and Shelf Life.

[63] Escobar M.A.M., Jaramillo F. (2015). Thermally and UV Stable Natural Dyes with Potential Use in Efficient Photoelectrochemical Devices. J. Renew. Mater..

[64] Mäkilä L., Laaksonen O., Kallio H., Yang B. (2017). Effect of processing technologies and storage conditions on stability of black currant juices with special focus on phenolic compounds and sensory properties. Food Chem..

[65] Veronique T., Wing Y.M., Christian D. Chapter Seabuckthorn Polyphenols: Characterization, Bioactivities and Associated Health Benefits. www.intechopen.com.

[66] Zadernowski R., Naczk M., Czaplicki S., Rubinskiene M., Szałkiewicz M. (2005). Composition of phenolic acids in sea buckthorn (*Hippophae rhamnoides* L.) berries. J. Am. Oil Chem. Soc..

