# Supplementary material for: The Stability of Phenolic Compounds in Fruit, Berry, and Vegetable Purees Based on Accelerated Shelf-Life Testing Methodology
Source: Foods. 2023 Apr 25;12(9):1777. doi: 10.3390/foods12091777 (PMC10178123; doi:10.3390/foods12091777)
Supplement: Supplementary file 1 [file foods-12-01777-s001.zip › Supplementary Materials_Table S1.pdf]

Supplementary Materials

The Stability of Phenolic Compounds in Fruit, Berry, and Vegetable Purees Based on Accelerated Shelf-Life Testing Methodology

Kärt Saarniit*, Hanna Lang, Rain Kuldjärv, Oskar Laaksonen and Sirli Rosenvald

* Correspondence: kart@tftak.eu

Table S1. Changes in TPC (mg GAE/100 g) of four-grain puree with banana and blueberry (FGBB), mango-carrot-sea buckthorn puree (MCB), and fruit and yogurt puree with biscuit (FYB) during storage tests at 23°C and 40°C.

Time point (days)	Packaging	FGBB	MCB	FYB
		23°C		
0	AL-layered	30.9±1.7[a]	51.7±2.6[a]	49.8±1.5[ab]
	AL-free	29.2±1.5[a]	53.0±3.0[a]	50.0±1.5[ab]
182	AL-layered	19.1±0.4[b]	53.2±2.6[a]	52.6±2.6[a]
	AL-free	19.1±1.1[bc]	49.4±2.4[ab]	49.8±2.0[ab]
274	AL-layered	19.7±0.8[b]	51.3±1.1[bc]	51.0±4.2[ab]
	AL-free	18.6±1.6[b]	49.1±2.5[c]	49.1±3.9[b]
365	AL-layered	16.3±2.5[cd]	38.2±3.0[d]	40.1±4.8[c]
	AL-free	15.5±2.8[d]	36.7±3.1[de]	39.6±2.3[c]
427	AL-layered	11.4±1.7[e]	37.7±2.1[ef]	33.8±2.3[d]
	AL-free	10.2±2.4[e]	33.6±4.4[f]	31.3±2.1[d]
		40°C		
0	AL-layered	30.9±1.7[a]	51.7±2.6[a]	49.8±1.5[ab]
	AL-free	29.2±1.5[a]	53.0±3.0[a]	50.0±1.5[ab]
28	AL-layered	17.0±1.0[b]	51.7±3.1[a]	43.4±2.0[bc]
	AL-free	17.9±1.5[bc]	50.0±2.8[a]	44.0±1.8[bd]
42	AL-layered	12.3±1.2[cd]	50.1±4.4[a]	46.5±1.7[e]
	AL-free	12.7±1.2[d]	49.0±1.6[a]	45.2±0.9[ce]
56	AL-layered	15.2±0.6[cd]	52.0±2.1[a]	46.8±1.4[bce]
	AL-free	12.5±1.7[d]	51.0±1.6[a]	45.0±2.4[bce]
66	AL-layered	13.4±1.5[cd]	52.5±2.8[a]	41.4±1.8[df]
	AL-free	11.7±2.1[d]	49.0±1.6[a]	40.4±2.4[f]

The significances are calculated across time points and packaging for each different product and each different storage temperature by using pairwise t-test comparisons between the estimated marginal means.
